# Evolution of conditional cooperation under multilevel selection

**DOI:** 10.1038/srep23006

**Published:** 2016-03-11

**Authors:** Huanren Zhang, Matjaž Perc

**Affiliations:** 1 Social Science Division, New York University Abu Dhabi, P.O. Box 129188, Abu Dhabi, United Arab Emirates; 2 Faculty of Natural Sciences and Mathematics, University of Maribor, Koroška cesta 160, SI-2000 Maribor, Slovenia; 3 CAMTP – Center for Applied Mathematics and Theoretical Physics, University of Maribor, Krekova 2, SI-2000 Maribor, Slovenia

## Abstract

We study the emergence of conditional cooperation in the presence of both intra-group and inter-group selection. Individuals play public goods games within their groups using conditional strategies, which are represented as piecewise linear response functions. Accordingly, groups engage in conflicts with a certain probability. In contrast to previous studies, we consider continuous contribution levels and a rich set of conditional strategies, allowing for a wide range of possible interactions between strategies. We find that the existence of conditional strategies enables the stabilization of cooperation even under strong intra-group selection. The strategy that eventually dominates in the population has two key properties: (i) It is unexploitable with strong intra-group selection; (ii) It can achieve full contribution to outperform other strategies in the inter-group selection. The success of this strategy is robust to initial conditions as well as changes to important parameters. We also investigate the influence of different factors on cooperation levels, including group conflicts, group size, and migration rate. Their effect on cooperation can be attributed to and explained by their influence on the relative strength of intra-group and inter-group selection.

Scientists have identified direct reciprocity as one important explanation for the emergence of cooperation^1^. This principle can be roughly summarized as “do onto others what they did onto you”. Based on this principle, cooperation can be established as people give up short-term gain to achieve a higher return in the long run. The concept of direct reciprocity was introduced by^2^, using the term “reciprocal altruism”. One classical example of a strategy that exemplifies this principle is Tit-For-Tat. Tit-For-Tat starts with cooperation, then always copies the opponent’s move in the previous round. In Axelrod’s computer tournaments, where strategies were invited to play the prisoner’s dilemma repeatedly, this simple strategy submitted by Anatol Rapoport outperformed other much more complex strategies and became the winner of the tournaments^3^,^4^.

Axelrod summarizes three properties exhibited by successful strategies in the tournaments: niceness, forgiveness, and provocability. They are never the first to defect (nice); they forgive an isolated defection and are willing to come back to cooperation (forgiving); they are always provoked by a defection no matter how good the interaction has been so far (provocable). Axelrod also uses an evolutionary experiment with a population initially using the submitted strategies (and a strategy that randomly chooses an action), where an individual’s expected number of offspring is proportional to the average payoff they receive. Tit-For-Tat thus quickly becomes the most widely-used strategy, and the success implies that the principle of direct reciprocity (provocability and forgiveness) makes it possible for cooperation to be established^5^. Indeed, many other computational models have demonstrated that strategies incorporating direct reciprocity are vital for the emergence of cooperation^6^,^7^,^8^,^9^,^10^.

In human societies, many activities require collective endeavor with more than two people, making the two-player prisoner’s dilemma an inadequate model. Such situations can be modeled as a public goods game, an *n*-player extension of the prisoner’s dilemma^11^,^12^. In a public goods game, players decide how much of their endowment to contribute to the public good that benefits all group members. While it is socially optimal to contribute everything, players can get higher payoff if they contribute nothing, regardless of other people’s actions.

Using evolutionary games, it has been shown that spatial structure can give rise to different patterns that promote cooperation in comparison to a well-mixed population^13^,^14^,^15^,^16^,^17^,^18^,^19^. In the context of the public goods game, many mechanisms, including voluntary participation^20^, opportunity to select neighbors^21^,^22^, reward and punishment^23^,^24^,^25^,^26^,^27^,^28^,^29^, referring to the social performance^30^, and heterogeneity in the number of neighbors^14^, in wealth distribution^31^, in learning activities^32^, in benefit obtained from the public goods^33^,^34^, as well as the group size^35^,^36^,^37^ and in-group favoritism^38^,^39^ have been shown to promote cooperation.

Considering the similarity between prisoner’s dilemma and public goods games, a natural question to ask is whether direct reciprocity that works well in two-player prisoner’s dilemma can be generalized to a social dilemma that involves *n* players. In order for direct reciprocity to function effectively, players need to condition their actions on the actions of others so that free-riders can be punished. In public goods provision, however, it is hard, if not impossible, for direct reciprocity to work. Players can punish low performance by withdrawing contribution, but this punishment cannot be directed exclusively to free-riders – it hurts other cooperators as well. On the other hand, reducing contributions to punish free-riding can be misinterpreted as defecting. This ambiguity of intention makes it hard for conditional strategies to work, and in principle, the consensus is that the extension is problematic because of the inherently imperfect information in terms of with whom to reciprocate^40^. Nevertheless, recent research shows that some approaches, like image scoring, work very well in groups too^41^.

Several experiments have confirmed that many subjects in the lab do use conditional strategies in the context of public goods games^42^,^43^,^44^. Given its importance and prevalence, it is surprising that not much research has been devoted to studying conditional strategies in the realm of the public goods game. In^45^, for example, a set of six strategies with binary contribution levels was considered, and it was shown that the most cautious conditional strategy – contribute when everyone in the group contributes – is likely to dominate the population. Notably, punishment and reward can also be considered as conditional strategies. However, these require information on each player’s performance, which is often unavailable. In this paper, we show that with full anonymity and only information on the average contribution level, the use of conditional strategies alone can still make cooperation possible.

This paper extends previous work and strives to understand and explain how people’s conditional strategies came into existence in the presence of multilevel selection, and how the existence of conditional strategies influences cooperation. In contrast to previous studies, we consider continuous contribution levels and assume individuals use piecewise linear response functions. While linear response functions have been studies in the context of two-player alternating prisoner’s dilemma^46^,^47^,^48^,^49^, we found no records of their consideration in the context of the public goods game. This setup enables us to consider a wide range of conditional strategies with continuous contribution levels, which allows for interesting and rich interactions between strategies.

Multilevel selection imposes structure on the population, which can be considered as a kind of spatial structure^50^,^51^,^52^,^53^. While in most spatial/network models individuals are members in multiple groups, people can belong to only one group in multilevel selection model. While conventional spatial/network games can represent social interactions in a complex society, multilevel selection can characterize ancestral human societies where people closely interact with their group members within a tribe. Because multilevel selection model reflects the situation in early human societies where cooperative preferences are likely to evolve [see^54^, Chapter 6] for detailed discussion], it is an important model to understand human cooperation.

Multilevel selection has been shown to be promising to explain the large-scale cooperation that exists only in human societies^55^,^56^, and it has been extensively used in the literature to study the evolution of social institutions^57^, indirect reciprocity and social norms^58^,^59^, the emergence of altruistic punishment^60^,^61^,^62^, and parochial altruism^63^,^64^,^65^. Previous studies on the evolution of cooperation in the setting of multilevel selection mainly focus on unconditional strategies. One conclusion from these studies is cooperation is possible only when intra-group selection is weak and there is enough “reproductive leveling”^66^. This paper contributes to the literature of multilevel selection by considering conditional strategies, and we show that the introduction of conditional strategies make cooperation possible, even with strong within-group selection.

## Results

**Figure 1:**
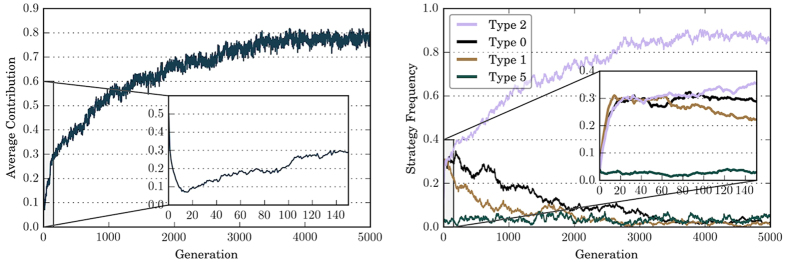
Average contribution and strategy frequencies over time, averaged over 50 independent runs. In the left panel, depicting the dynamics of the contribution level, it can be observed that the average contribution level drops from 0.5 to below 0.1 during the first 20 generations, then gradually increases and eventually stabilizes around 0.8. In the right panel, depicting the evolution of strategies, it can be observed that among all 27 strategies, only 4 account for non-negligible proportions in the population. We first observe steep increases in the numbers of Type 0, Type 1, and Type 2, which coincides with the decrease in contribution level. After the first 20 generations, Type 2 gradually increases in number and eventually dominates in the population. The spread of Type 2 in the population is accompanies by the increase in the average contribution level. Strategies with average frequency less than 3% are excluded from the graph for clarity.

The dynamics of contribution level and the evolution of strategies using baseline parameter values are shown in Fig. 1 [see Methods for the evolutionary model and the representation of strategies, and Table 1 for parameters]. In the left panel of Fig. 1, we see that the average contribution starts from 0.5, which is consistent with the contribution level when individuals all choose a random strategy. During the first 20 generations, contribution drops to below 0.1. After around generation 20, the average contribution level gradually increases and eventually reaches and stabilizes around 0.8. In contrast, if we constrain the strategy space to unconditional strategies, cooperation cannot be established: Type 0 will quickly dominate in the population, and the contribution level gets stuck around 0 throughout the simulation (not shown). This shows that conditional strategies play an important role in the emergence of cooperation. Notably, if we only consider unconditional strategies, individual selection must be weak (e.g., *w* > 10) in order for cooperation to emerge.

The right panel of Fig. 1 demonstrates that, among all the 27 strategies, only 4 account for non-negligible proportions in the population. At the start, each of the 27 strategies is used by a bit more than 3% of the population, then we observe steep increases in the numbers of three strategies during the first 20 generations – Type 0, Type 1, and Type 2. After around 20 generations, Type 2 gradually increases in number and its frequency eventually stabilizes around 90%.

Comparing the left and the right panel of Fig. 1, we see that the initial decrease in cooperation level coincides with the increase in the number of Type 0, Type 1, and Type 2 in the population, while the stable increase is accompanies by the increase in the number of Type 2 strategy.

**Figure 2:**
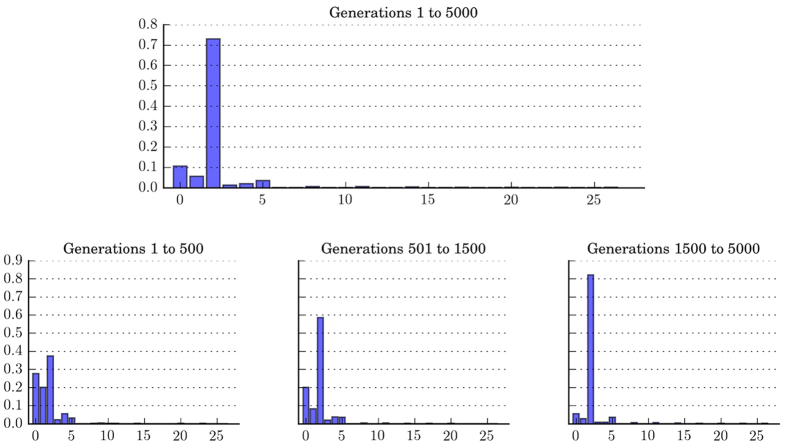
Strategy distribution over time. Initially (Generations 1 to 500), Type 0, Type 1, and Type 2 all account for significant proportions. However, Type 2 eventually dominates in the population. Type 5 (perfectly conditional cooperators) accounts for a more or less constant but small proportion throughout the span of simulation. Data is averaged over 50 runs of simulation.

Figure 2 provides the histograms showing the frequencies of all 27 strategies in different time of the evolution. Initially, Type 0, Type 1, and Type 2 all account for significant proportions in the population, but eventually Type 2 becomes the predominant strategy.

What drives the changes in the cooperation level and strategy composition? As illustrated by Equation (4), there is a tension between inter-group selection and intra-group selection: while strong inter-group selection tends to increase cooperative behavior, intra-group selection drives it away. It is therefore interesting to investigate and compare the relative strength for these two types of selection.

Due to the large number of possible strategies considered here, it provides limited insight if we calculate and compare the variance of each strategy’s frequency. Instead, we can simply use the variations in contribution level, E[Var(*c*_*ij*_)] and Var(*c*_*j*_), as proxies for the strength of intra-group and inter-group selection [see Methods for details].

**Figure 3:**
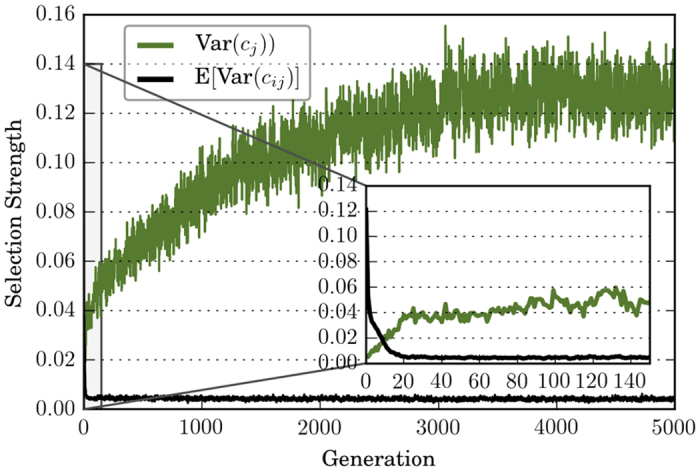
Intra-group and inter-group selection strength over time, averaged over 50 runs. E[Var(*c*_*ij*_)], proxy for the strength of intra-group selection, starts around 0.12, then quickly drops to and stabilizes around 0.04 during the first 20 generations. In contrast, Var(*c*_*j*_), proxy for the strength of inter-group selection, starts near 0, steadily increases, and eventually stabilizes around 0.12. In comparison with the left panel of Fig. 1, the decrease in the average cooperation level coincides with the time span where intra-group selection is strong relative to inter-group selection. After the strength of intra-group selection drops and stabilizes, intensifying inter-group selection causes the average cooperation level to increase.

Figure 3 demonstrates the strength for intra-group and inter-group selection over time. E[Var(*c*_*ij*_)] starts around 0.12, then quickly drops to and stabilizes around 0.04 during the first 20 generations. In contrast, Var(*c*_*j*_) starts near 0, steadily increases, and eventually stabilizes around 0.12. Comparing Fig. 3 with the left panel of Fig. 1, we see that the decrease in the average cooperation level coincides with the time span where intra-group selection is strong relative to inter-group selection. After the strength of intra-group selection drops and stabilizes, stronger inter-group selection causes the average cooperation level to increase.

The dynamics of selection strength sheds light on the characteristics of successful strategies. With strong intra-group selection, we observe increases in the numbers of the three strategies: Type 0, Type 1, and Type 2. All these three strategies are characterized by zero contribution when 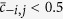. In an environment where everyone randomly chooses a strategy, individuals using these strategies cannot be exploited and can sometimes take advantage of other group members’ contribution. For the two conditionally cooperative strategies Type 1 and Type 2, they never contribute more than 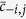. These observations lead us to conclude that unexploitable strategies can persist in the population when intra-group selection is strong.

As intra-group selection subsides and inter-group selection intensifies, Type 2 starts to dominate in the population. Compared to Type 1, groups with all Type 2 individuals can achieve full contribution. Because of this, this strategy eventually drives out Type 1 in the presence of strong group selection. This provides evidence that strategies that make full contribution possible on the group level will spread and dominate in the population, even when inter-group selection is strong.

In summary, Type 2 becomes the predominant strategy due to its two important properties: 1. It cannot be exploited by other strategies on the intra-group selection; 2. It makes high group contribution possible on the group level. Type 1 and Type 0 perform well in the presence of intra-group selection, but they cannot sustain high contribution and will lose during inter-group competition.

Type 5 (perfectly conditional cooperators) seems to be a great candidate for successful strategies, because it can achieve a high group contribution level, and at the same time they never contributes more than the average contribution level. However, it only account for a quite small (although stable) proportion throughout the evolutionary process. This is because it does not have the second property of Type 2. When “irrational” strategies, such as always cooperate (Type 26) or cooperate when average contribution is low (Type 18), are present in the population due to random mutation, they will induce Type 5 to contribute. Free-riders in situations like this would outperform Type 5 and take over the population. Type 2 on the other hand is resistant against these irrational strategies and can survive when intra-group selection is strong.

### Effect of initial conditions

**Figure 4:**
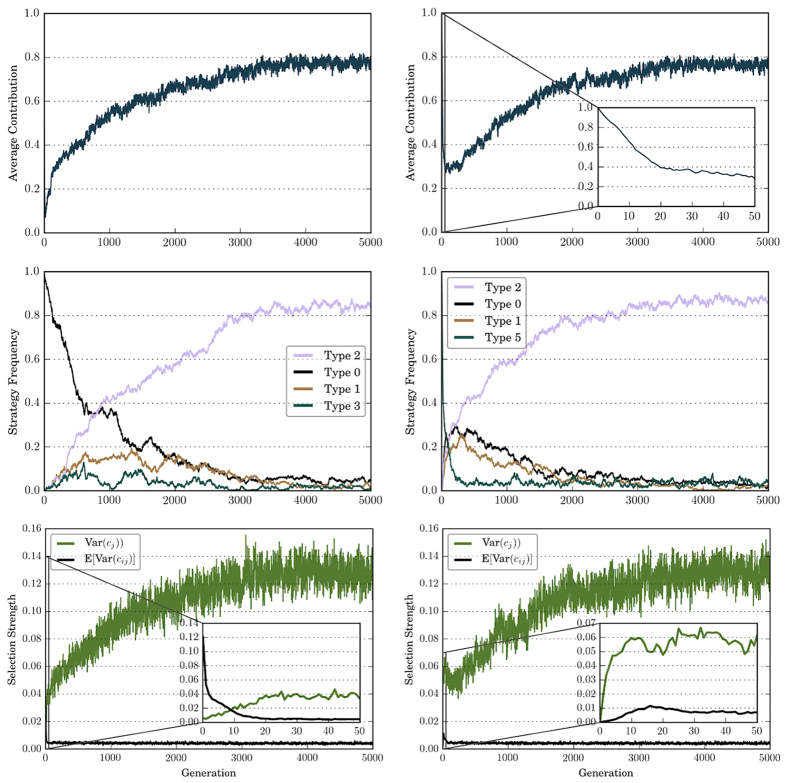
Dynamics of contribution, strategy frequency, and selection strength when the population is initialized as Type 0 (left column of panels) or Type 5 (right column of panels). With different initial conditions, average contribution, strategy frequency, and selection strength all eventually converge to similar levels, and the success of Type 2 is robust to initial conditions. Top panels: average contribution level eventually stabilizes around 0.8. Middle panels: Type 2 eventually dominates in the population. Bottom panels: strength of intra-group selection and strength of inter-group selection stabilize around similar levels with different initial conditions.

To test the robustness of Type 2 strategy against different initial conditions, we run simulations where population are initialized with different strategies. Figure 4 shows the dynamics when the population is initialized as all free-riders (Type 0) or all perfectly conditional cooperators (Type 5). We see that the population initialized with all free-riders exhibit similar dynamics as the population randomly initialized: Var(*c*_*j*_) quickly drops from 0.14 and stabilizes around 0.004, while E(Var(*c*_*ij*_)) gradual increases. With the intensifying inter-group-level selection, we also observe an increase in the contribution level and the number of Type 2 strategies. When the population starts with all perfectly conditional cooperators, we first observe a sharp reduction on the average contribution level and then a gradual increase. There is also a first decrease and then increase in the strength of inter-group selection.

In both initial conditions, as in the case with random strategies, the contribution level eventually stabilizes around 0.8. As for the selection strength, it eventually stabilizes around 0.004 on the individual level and around 0.12 on the group level. Further investigation shows that Type 2 will eventually become the predominant strategy if we start with homogeneous population with other strategies — the initial conditions do not affect the success of Type 2 strategy, nor do they influence the long-run contribution level, strategy frequency, or selection strength.

### Effects of group conflict, group size, and migration

**Figure 5:**
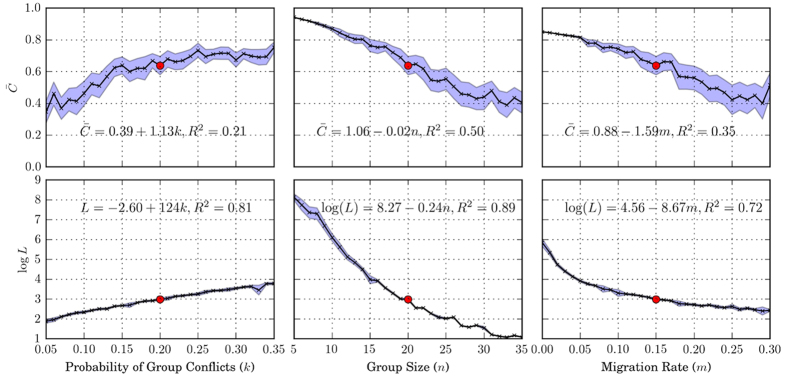
Average cooperation level 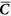 and the logarithm of the average length of cooperative epochs log(*L*) for different parameter values. Shaded area represents the corresponding 95% confidence intervals. The values of 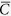 and log(*L*) obtained using baseline parameters are marked as red dotes. Comparing graphs on the same row can give us some idea on the relative effects of different factors on cooperation (and its stability). Regressions of the dependent variable on the parameter of interest are also shown in the figures. As shown in the graphs, cooperation emerges for a wide range of parameter values. Increasing the probability of group conflicts, decreasing group size, or decreasing migration rate all lead to an increase in cooperation level and stability of cooperation. In all simulations where cooperation levels are high, we observe the prevalence of Type 2 strategy.

Figure 5 illustrates how cooperation changes as we vary the probability of group conflicts (*k*), group size (*n*), and migration rate (*m*). Besides average contribution level, we also use the average length of cooperative epochs to measure the stability of cooperation quantitatively. Cooperative epochs are defined as sustained periods where population contribution levels are above 60%. We note that the presented results are insensitive to the threshold used in the definition of cooperative epochs.

Increasing the probability of group conflicts, decreasing group size, or decreasing migration rate can all lead to an increase in the cooperation level and stability of cooperation. How cooperation changes with different parameters can be explained by the tension between group-level selection and individual-level selection. Effects that increase Var(*c*_*j*_) tend to increase cooperation level, while effects that increase E[Var(*c*_*ij*_)] tend to decrease cooperation. Because group conflicts intensify inter-group selection, we see cooperation level increases with the probability of group conflicts. On the other hand, increasing group size or migration rate decreases cooperation. Figure 5 also gives the expressions for average cooperation level and average length of cooperative epochs as a function the corresponding parameters.

As shown in Fig. 5, cooperation can emerge for a wide range of parameter values. For all the parameters investigated in simulations, we observe that high cooperation levels are always accompanied by the prevalence of Type 2 strategies. This also provides evidence on the robustness of Type 2.

### Effect of selection strength

**Figure 6:**
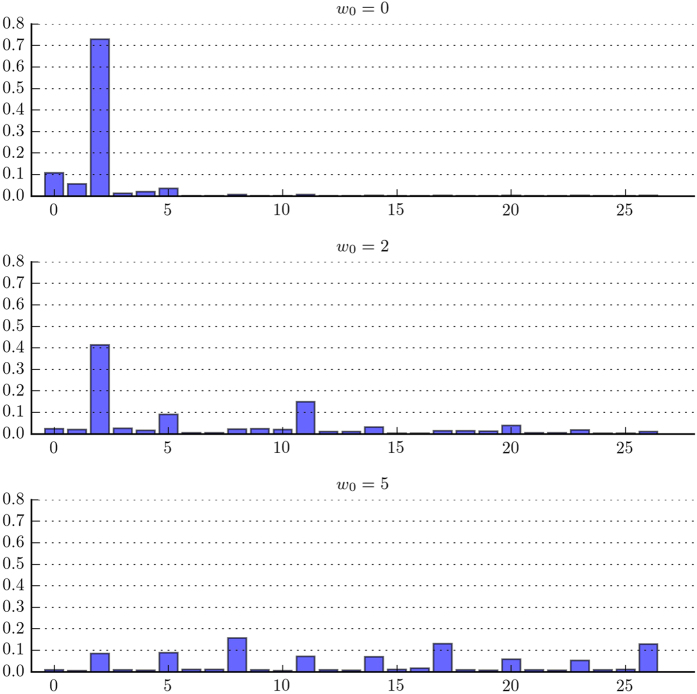
Average cooperation level 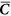 and the logarithm of the average length of cooperative epochs log(*L*) for different baseline fitness (*w*_0_). Shaded area represents the corresponding 95% confidence intervals. As shown in the graphs, cooperation emerges for a wide range of parameter values. Increasing the probability of group conflicts, decreasing group size, or decreasing migration rate all lead to an increase in cooperation level and stability of cooperation. In all simulations where cooperation levels are high, we observe the prevalence of Type 2 strategy. Overall cooperation level increases with the value of *w*_0_: contribution increases from a bit over 60% at *w*_0_ = 0 to over 80% at *w*_0_ = 5. The stability of cooperation does not change significantly with selection strength.

To investigate the effect of selection strength on the evolution of conditional cooperation, we run the simulation for different values of baseline fitness *w*_0_. We know that in the case of unconditional cooperation, greater *w*_0_ indicates weaker intra-group selection, making it easier for cooperation to get established. Figure 6 shows that increasing the value of *w*_0_ can indeed increase overall cooperation level. As shown in the top panel, overall contribution level increases from a bit over 60% at *w*_0_ = 0 to over 80% at *w*_0_ = 5. The stability of cooperation does not change significantly with selection strength, as shown in the bottom panel.

**Figure 7:**
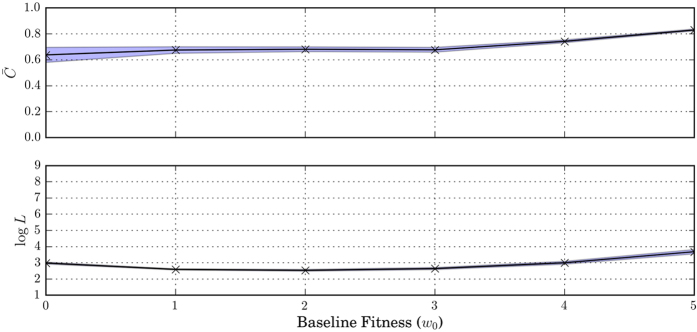
Distributions of strategies for different values of baseline fitness (*w*_0_). As we increase *w*_0_, strategies that prescribe cooperation even when group contribution is low can persist, because there is little selection against these strategies. Because of group-level selection, all the strategies that persist in the population prescribe full contribution when all the group members cooperate.

When intra-group selection is weak enough, even unconditional cooperation can emerge. Therefore, we would expect that unconditional cooperation can persist in the population with large *w*_0_. This is indeed the case, as shown in Fig. 7. As we increase *w*_0_, strategies that prescribe cooperation even when group contribution is low start to persist in the population: there is little selection against these strategies with large *w*_0_ because cooperation level is often high. Due to group-level selection, however, all the strategies that persist in the population prescribe full contribution when all the group members cooperate.

## Discussion

In this paper, we use the concept of multilevel selection to study the emergence of conditional cooperation in the setting of public goods provision. We consider a strategy space with 27 possible conditional strategies represented by piecewise linear response functions, which allows a wide range of possible interactions between strategies. Under full anonymity, players only know the average group contribution level, but not the contribution of other group members. Our results may help explain the emergence of cooperation without mechanisms that requires individual information, such as reputation and punishment.

The existence of conditional strategies renders cooperation a viable and stable alternative. In contrast, if people are constrained to use unconditional strategies, cooperation can not prevail under intra-group selection. The strategy that eventually dominates in the population is Type 2, which can achieve full group cooperation but at the same time is unexploitable by others. Changing the probability of group conflicts, group size, or migration rate does not influence the success and prevalence of Type 2 strategy, thus indicating notable robustness of this evolutionary outcome.

In our model, we have a wide range of conditional strategies. One potential concern is whether the population size (400) is large enough to allow for interactions between this large number of strategies. To address this concern, we run simulations for different population size while keeping the group size fixed at 20. We observe no significant differences in the overall cooperation levels and the strategy distributions, indicating that the population size used is sufficiently large for investigating the evolution of 27 strategies [see Supplementary Information for details].

As pointed out before, the multilevel selection model assumes that individuals always play against the same group of people. While this captures some characteristics of early human society, it would be interesting to investigate different network structures where individuals can interact with different groups people. Future research can introduce continuous response function to different population structures, which may provide deeper insight into the emergence of conditional cooperation.

## Methods

Consider a population with *g* groups, each group *j* has *n*_*j*_ members (*j* = 1, 2, …, *g*). Each individual has initial endowment 1. Individual *i* in group *j* decides the amount *c*_*ij*_ (0 ≤ *c*_*ij*_ ≤ 1) to contribute to a public good that benefits all members in the same group. Contribution by all group members will be multiplied by *r* and then evenly distributed among all group members. The payoff of individual *i* in group *j* can be expressed as


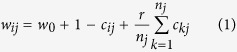


where 1 < *r* < *n*_*j*_ is the total return to cooperation, and 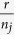 is the Marginal Per Capita Return (MPCR). The first term *w*_0_ represents the baseline payoff, including other factors that can influence players’ payoffs but not captured by public goods provision.

It is optimal for the group if everyone contributes all their endowment. However, because the MPCR is less than 1, individuals can always be better off by contributing less to the public good, no matter what other members do. The only Nash equilibrium in this game is therefore zero contribution by all members.

Analysis from evolutionary game theory also shows that any individual making positive contribution will eventually be weeded out by the evolutionary process. In evolutionary game theory, the transmission of a trait (or a behavior, a strategy) is related to the payoff of the individual with that trait. Notably, the transmission can be genetic or cultural. In genetic transmission, genes bring higher payoffs are more likely to pass on; in cultural transmission, successful strategies are more likely to be learned by others. As the nature of the transmission does not influence the theoretical implications, we can abstract from the underlying mechanism of transmission of traits. When *w*_0_ in Equation (1) is small relative to 1, the selection on this trait is strong. If *w*_0_ is relatively large, we have weak selection. It is obvious to see that the intra-group selection will eventually drive out cooperative individuals, whether it is strong or weak selection. However, if there is inter-group selection that is strong enough compared to intra-group selection, cooperation can get established.

### The Price equation

The viability of cooperation under multilevel selection can be illustrated using the Price equation^67^. The Price equation assumes selective migration where groups with higher average payoffs will grow in size compared to those with lower payoffs. Denote *q*_*j*_ as the fraction of the population that is in group *j*. Let *w*_*j*_ = ∑_*i*_*w*_*ij*_/*n*_*j*_ be the average payoff group *j*, and *w* = ∑_*j*_*q*_*j*_*w*_*j*_ be the average population payoff.

The sizes of the groups change from one period to the next proportionally to their relative payoff. Denote 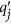 as the fraction of the population in group *j* in the next period, then selective migration prescribes


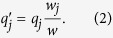


Now consider the altruistic trait *A* which prescribes full contribution to the public good, and the selfish trait *S* which prescribes zero contribution. The Price Equation can be used to study any trait that can potentially influence individual payoffs. Because a cooperative action by definition benefits others at a personal cost, here cooperation and altruism are used interchangeably and we can thus simply call individuals with trait *A* (unconditional) cooperators. We use *p*_*ij*_ = 1 to represent that individual *i* in group *j* has the trait *A* and *p*_*ij*_ = 0 otherwise. Denoting *p*_*j*_ as *A*’s frequency in group *j* and *p* its population frequency of *A*, we can derive the following Price equation [refer to Supplementary Information for details]


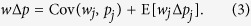


Here the expectation and covariance are weighted by *q*_*j*_. Because *w* is always positive, trait *A* increases in frequency as long as the right hand side is positive. The change in its population frequency can hence be partitioned into two parts: the two terms on the right hand size characterize inter-group and intra-group selection respectively.

Substituting the payoff represented by Equation (1) into the above equation, we obtain the Price equation in the context of public goods provision:


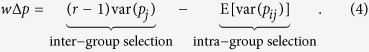


Note that the second term is always negative. In order for evolution to favor altruists, we need inter-group selection measured by var(*p*_*j*_) to be strong compared to intra-group selection measured by E[var(*p*_*ij*_)]. Assuming individuals are drawn to reproduce with probability equal to their share of the total group payoff, we must have *w*_0_ ≫ 1 in Equation (1) to make it possible for cooperation to emerge. With strong selection *w* ≈ 0, the payoff of a defector in a group of cooperators is much higher, making it impossible for trait *A* to persist in the population.

The Price equation has great limitations. It only applies to systems with a relatively small number of traits (strategies). As we include more strategies, it quickly becomes intractable to characterize the system analytically. It helps identify equilibria but cannot provide the dynamics that lead to the equilibria. We know that group conflicts caused by limited resources were common in early human society, but the Price equation is abstracted away from direct group conflicts – considering selective extinction (where groups become extinct due to conflicts or natural disasters) makes it difficult to derive closed-form solutions. To overcome these limitations, we use agent-based modeling to provide insight into systems with interactions between a broad set of conditional strategies. In order to add realism to our model, instead of using Equation (2) to model group-level selection, we introduce direct group conflict; we also incorporate migration between groups into the evolutionary model.

### Representation of conditional strategies

Before delving into the details of agent-based simulation, we will first discuss how conditional strategies are represented.

Experiments show that many people are conditional cooperators — they condition their contribution to the public good on other people’s contribution level. To capture this experimental regularity, we use a response function to represent conditional strategies. Denoting 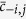 as the average contribution level by the other group members, we can represent *c*_*ij*_ as a function of 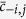.

**Figure 8:**
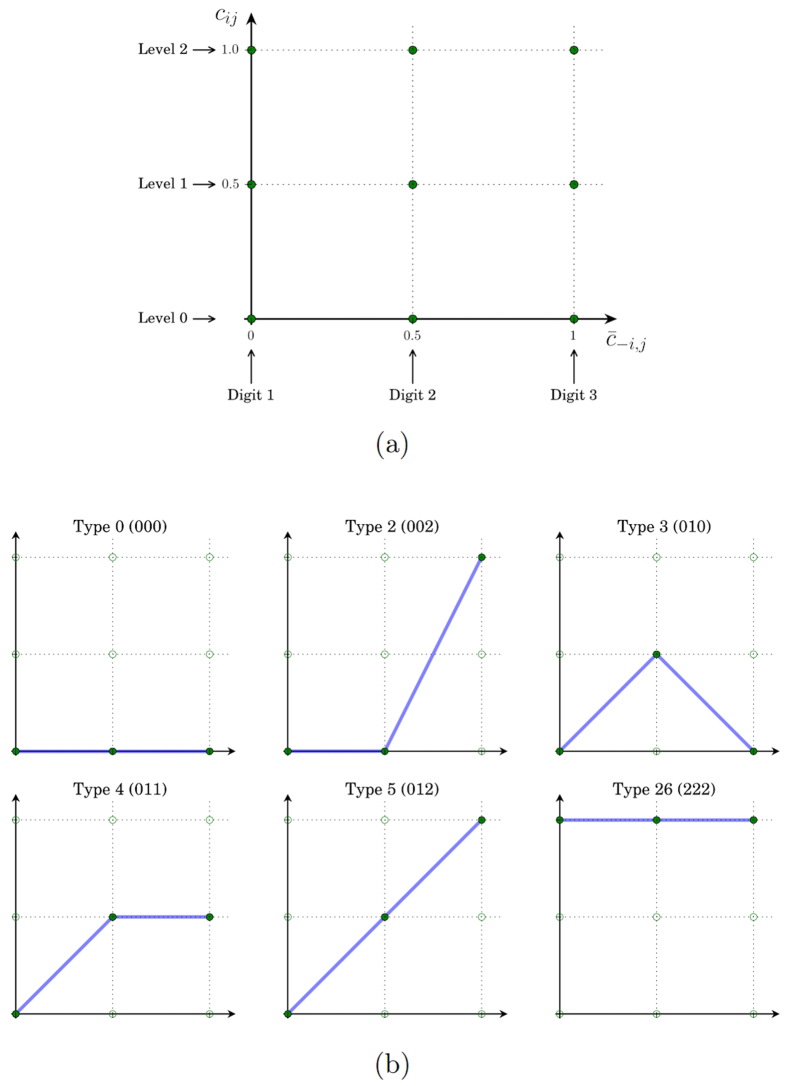
Representation of conditional strategies. Conditional strategies specify the contribution levels (*c*_*ij*_ ∈ [0, 1]) as functions of the average contribution level by the other group members (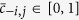). (**a**) Strategies are represented as linear response functions that are determined by three points: the contribution levels when 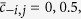 and 1, respectively. A response function is obtained by connecting those three points, and can be identified by a ternary string with length 3. (**b**) Examples of conditional strategies and their ternary string representations.

The contribution level must lie in [0, 1], so the response function is a mapping [0, 1] → [0, 1]. In order to keep the set of strategies relatively small and at the same time maintain flexibility, we assume all individuals use conditional strategies represented by piecewise linear response functions. As illustrated in Fig. 8, the response function is unique identified by three points: the contribution levels when 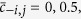 and 1, respectively. The response function is obtained by connecting those three points.

At 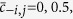 or 1, *c*_*ij*_ can take three possible values 0, 0.5, 1. Each strategy can therefore be identified by a ternary string with length 3. Each digit of the string takes three possible values 0, 1, 2, representing zero contribution, medium contribution (0.5) and high contribution (1) respectively.

As an example, suppose a strategy prescribes a contribution level of 0.5 when 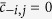, then the first digit of the ternary string will be 1. Suppose it further prescribes contribution levels of 0 and 1 for 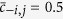 and 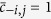 respectively, then the second and third digit of the ternary string are 0 and 2. Connecting these three points (0, 0.5), (0.5, 0), (1, 1), we form the piecewise linear response function represented by the ternary string 102.

The 27 strategies considered in this paper are identified by the decimal number converted from their ternary strings. Figure 8 gives some examples of common conditional strategies represented as piecewise linear response functions. For example, a Type 0 (000) is a free rider who always contributes 0, a Type 5 (012) is a perfectly conditional cooperator who always contributes the average contribution level by the other group members, and a Type 26 (222) is an unconditional cooperator who never contributes anything. A Type 2 (002) contributes nothing when the average contribution by other group members is less than 0.5, but gradually increases its contribution when the average contribution increases from 0.5; when the average contribution reaches 1, it also contributes 1.

In order to make conditional strategies possible, interactions between group members need to be repeated. We assume the contribution level by each individual is the equilibrium contribution level given all players’ strategies: everyone’s belief is the same as the actual average contribution by other group members, and everyone contributes according to her strategy and her belief. The equilibrium contribution level is simply the average contribution level when interactions are repeated infinitely. During the interactions, individuals constantly adjust their beliefs according to the actual contributions and adjust their contributions level based the updated beliefs. When none of the group members change their beliefs and contributions, contributions reach equilibrium.

Under certain circumstances, initial beliefs may influence the equilibrium contribution. When every group member uses Type 5 (perfectly conditional cooperation), for example, the equilibrium contribution will be the same as the initial belief. In the simulation, we assume individuals start with optimistic beliefs – they believe everyone will exert full contribution initially – and adjust their beliefs and contribution in the process of interacting with others.

### Agent-based simulations

**Figure 9:**
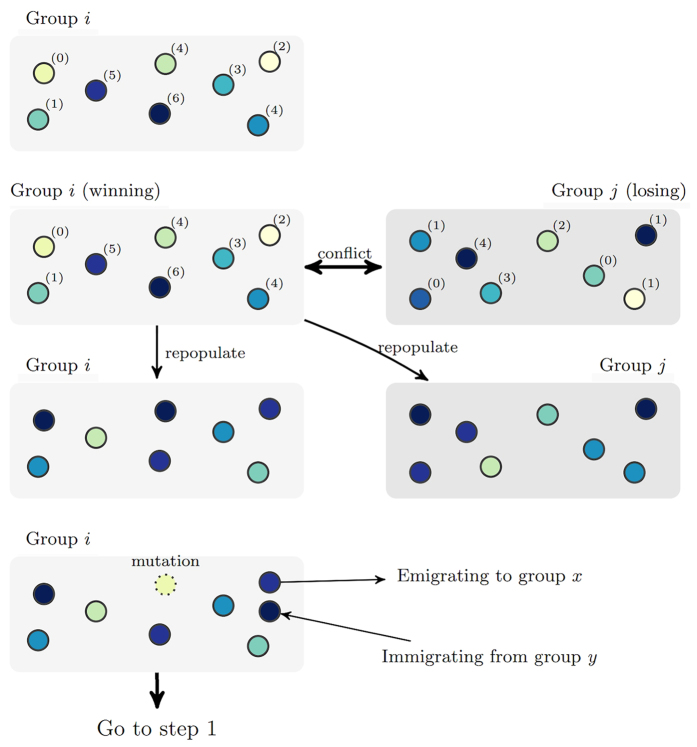
Evolution of conditional strategies with multi-level selection. From top to bottom, the following steps are depicted. (i) *Public goods provision*. Agents contribute to the public goods according to their strategies and receive payoffs. (ii) *Group competition*. With probability *k*, groups engage in conflict. Groups in conflict are randomly paired. The probability for group *i* to win against its paired group *j* in a conflict is 0.5 + 5(*C*_*i*_ − *C*_*j*_), where *C*_*i*_ represent the average contribution of group *i*. The winning group repopulates the site of a losing group during the reproduction phase. (iii) *Reproduction*. Replicas of the current generation constitute the next generation. They are reproduced by drawing (with replacement) from the current group membership with the probability equal to their share of the total group payoff. (iv) *Mutation*. With probability *μ*, an individual randomly chooses a strategy in the strategy space (with 27 possible strategies). (v) *Migration*. With probability *m*, an individual relocates to another randomly selected group.

Figure 9 demonstrates the evolutionary process of the agent-based simulation. The total population size and group size are kept constant throughout the simulation. Initially, each individual uses a random strategy. Individuals then reproduce and pass on their strategies based on the payoff each strategy brings. Only individuals reproduce, but the selection exists both on intra-group level and inter-group level. Strategies that perform well within a group are likely to spread within the group, while strategies that make groups successful are more likely to prevail among groups. During the group competition step, groups with higher contribution levels are more likely to win if a conflict happens. When the difference between the average contributions between two competing group is greater than 0.1, the group with higher contribution will win for sure. Note that here the difference in the average group payoffs is the same as the difference in the average group contributions, so equivalently, we can express the probability of winning as a function of the average group payoff. Because of this, we observe a tension between intra-group selection and inter-group selection. While strategies prescribing higher contribution are less advantageous within a group, groups with higher contribution are more likely to win in case of a conflict.

Assuming strong intra-group selection, the baseline payoff *w*_0_ specified in Equation (1) is set as 0. Mutation rate is set to be 0.01. In the baseline simulation, the population has 20 groups, each with *n* = 20 members. We also investigate the effect of probability of group conflict *k*, group size *n*, migration rate *m*, as well as the baseline fitness *w*_0_. Population size is kept constant around 400 as the group size *n* varies. The parameters explored in the simulation are shown in Table 1. How well these parameters reflect early human environments is discussed in^57^. For each set of parameters we have 50 runs of simulation, and each run lasts for *T* = 5000 generations. Because there is a large amount of randomness caused by group conflicts, reproduction, mutation, migration, etc., the results presented in the next section are based on the average over the 50 runs.

## Additional Information

**How to cite this article**: Zhang, H. and Perc, M. Evolution of conditional cooperation under multilevel selection. *Sci. Rep.* **6**, 23006; doi: 10.1038/srep23006 (2016).

## Supplementary Material

Supplementary Information

## Figures and Tables

**Table 1:** Values of the key parameters used during the simulations.

	Baseline values	Range explored
Group size (*n*)	20	5–30
Migration rate (*m*)	0.15	0.05–0.25
Probability of conflict (*k*)	0.20	0.05–0.30
Baseline fitness (*w*_0_)	0	0–5

According to^57^, these values might properly reflect early human environments.
