# Supplementary material for: Evolution of conditional cooperation under multilevel selection
Source: Sci Rep. 2016 Mar 11;6:23006. doi: 10.1038/srep23006 (PMC4786827; doi:10.1038/srep23006)
Supplement: Supplementary Information [file srep23006-s1.pdf]

Supplementary information: Evolution of conditional cooperation under multilevel selection

Huanren Zhang[1] and Matjaž Perc[2,3]

[1]*Social Science Division, New York University Abu Dhabi,*

P.O. Box 129188, Abu Dhabi, United Arab Emirates

[2]*Faculty of Natural Sciences and Mathematics,*

University of Maribor, Koroška cesta 160, SI-2000 Maribor, Slovenia

[3]*CAMTP – Center for Applied Mathematics and Theoretical Physics,*

University of Maribor, Krekova 2, SI-2000 Maribor, Slovenia

Derivation of the Price Equation

Consider a population with g groups, and the fraction of group j in the population is denoted as q_j. We note that the original Price Equation does not consider multilevel selection. The conclusion derived below is general enough to be applied to both a population with g individuals and a population with g groups (and there are individuals in each group). Let $w_j = \sum_i w_{ij}/n_j$ be the average payoff of group j, and $w = \sum_j q_j w_j$ be the average population payoff.

Denote q'_j as the fraction of the population in group j in the next period, then selective migration prescribes

$$q'_j = q_j \frac{w_j}{w}.$$

Consider a trait that can potentially influence the payoff of an individual. Let $p_{ij} = 1$ represent that individual i in group j has the trait and $p_{ij} = 0$ otherwise. Let p_j be the frequency of the trait in group j and p be its population frequency.

Denote p'_j and p' as the next-period average frequencies of the trait in group j and in the popu-

lation, then $p' = \sum_j q'_j p'_j$, and

$$\begin{aligned}\Delta p = p' - p &= \sum_j q'_j p'_j - \sum_j q_j p_j \\ &= \sum_j q_j \frac{w_j}{w}(p_j + \Delta p_j) - \sum_j q_j p_j \\ &= \sum_j q_j (\frac{w_j}{w} - 1) p_j + \sum_j q_j \frac{w_j}{w} \Delta p_j \\ &= \frac{1}{w} \sum_j q_j (w_j - w) p_j + \frac{1}{w} \sum_j q_j w_j \Delta p_j \\ &= \frac{1}{w} [\mathrm{Cov}(w_j, p_j) + \mathrm{E}(w_j \Delta p_j)].\end{aligned}$$

The last line uses the fact that $\mathrm{Cov}(w_j, p_j) = \sum_j q_j (w_j - w) p_j = \sum_j q_j (w_j - w)(p_j - p)$. This gives rise to the Price equation

$$w\Delta p = \mathrm{Cov}(w_j, p_j) + \mathrm{E}(w_j \Delta p_j).$$

In the specific case that $p_j = w_j$ (i.e., fitness itself is the characteristic of interest), the Price equation reformulates Fisher's fundamental theorem of natural selection: the rate of increase in fitness of any organism at any time is equal to its genetic variance in fitness at that time [1].

Evolution of altruism

Individuals in a group decide whether to contribute to the public good that can benefit all group members. Those with trait A will contribute 1 to confer a total benefit of r to the group. Using the notation from the main text, we have

$$w_{ij} = w_0 + 1 + r p_j - p_{ij}. \tag{I1}$$

We have the following relationships between individual quantity and group average quantity:

$$w_j = \frac{1}{n_j} \sum_i w_{ij},$$

$$p_j = \frac{1}{n_j} \sum_i p_{ij}.$$

Summing over all the individuals in group j for Equation I1, we obtain

$$w_j = w_0 + 1 + (r - 1) p_j.$$

Summing the above equation over all the group, we obtain

$$w = w_0 + 1 + (r-1)p.$$

To simplify the Price equation in the main text and rewrite the right hand side as functions of only p_{ij}, note that

$$w_{ij} - w_j = -(p_{ij} - p_j),$$

and

$$w_j - w = (r-1)(p_j - p).$$

Hence we can rewrite

$$\begin{aligned} \mathrm{cov}(w_j, p_j) &= \sum_j q_j (w_j - w)(p_j - p) \\ &= (r-1)\sum_j q_j (p_j - p)^2. \end{aligned}$$

This gives

$$\mathrm{cov}(w_j, p_j) = (r-1)\mathrm{var}(p_j). \tag{I2}$$

Notice that the replicas each individual has is proportional to its fitness level compared to the average fitness level of the group. Although the population fraction of group j may change in the next period, but because the fraction of A in group j does not depend on q_j or q_j', we can ignore the change in q_j when calculating p_j', and we have (to take into account the change in population fraction, we simply need to put q_j to both the numerator and the denominator of the expression, which keeps the result unchanged)

$$\begin{aligned} p_j' &= \frac{\sum_i \frac{w_{ij}}{w_j} p_{ij}}{\sum_i \frac{w_{ij}}{w_j}} \\ &= \frac{\sum_i w_{ij} p_{ij}}{\sum_i w_{ij}} \\ &= \frac{\sum_i w_{ij} p_{ij}}{n_j w_j}, \end{aligned}$$

and

$$\begin{aligned}\Delta p_j = p'_j - p_j &= \frac{\sum_i w_{ij}p_{ij}}{n_j w_j} - \frac{1}{n_j}\sum_i p_{ij} \\ &= \frac{1}{w_j}\frac{1}{n_j}(\sum_i w_{ij}p_{ij} - w_j \sum_i p_{ij}) \\ &= \frac{1}{w_j}\frac{1}{n_j}(\sum_i (w_{ij} - w_j)p_{ij}) \\ &= \frac{1}{w_j}\frac{1}{n_j}(\sum_i -(p_{ij} - p_j)p_{ij}).\end{aligned}$$

Therefore,

$$\begin{aligned}w_j\Delta p_j &= -\frac{1}{n_j}(\sum_i (p_{ij} - p_j)p_{ij}) \\ &= -\mathrm{var}(p_{ij}).\end{aligned}$$

We can then rewrite

$$\mathrm{E}(w_j\Delta p_j) = -\overline{\mathrm{var}}(p_{ij}). \tag{I3}$$

Substitute Equations (I2) and (I3) into the Price equation, we get

$$w\Delta p = (r-1)\mathrm{var}(p_j) - \overline{\mathrm{var}}(p_{ij}). \tag{I4}$$

Define the variance ratio R_V as the ratio of the between-group variance and the population variance

$$R_V = \frac{\mathrm{var}(p_j)}{\overline{\mathrm{var}}(p_{ij}) + \mathrm{var}(p_j)}. \tag{I5}$$

It is readily to see that the fraction of altruists p is stationary in the population when

$$R_V = \frac{1}{r}.$$

It is interesting to see that R_V measures the difference between the probability of being paired with an altruist conditional on being an altruist $P(A|A)$ and conditional on being a non-altruist $P(A|N)$, which means

$$R_V = P(A|A) - P(A|N).$$

To see this, notice that

$$P(A|A) = \frac{P(A,A)}{P(A)} = \frac{\sum_j q_j p_j^2}{p}.$$

Similarly

$$P(A|N) = \frac{P(A,N)}{P(N)} = \frac{\sum_j q_j p_j (1-p_j)}{1-p}.$$

Hence

$$\begin{aligned} P(A|A) - P(A|N) &= \frac{\sum_j q_j p_j^2}{p} - \frac{\sum_j q_j p_j (1-p_j)}{1-p} \\ &= \frac{(1-p)\sum_j q_j p_j^2 - p\sum_j q_j p_j(1-p_j)}{p(1-p)} \\ &= \frac{\sum_j q_j p_j^2 - p\sum_j q_j p_j^2 - p(p - \sum_j q_j p_j^2)}{p(1-p)} \\ &= \frac{\sum_j q_j p_j^2 - p^2}{p(1-p)} \\ &= \frac{\mathrm{E}(p_j^2) - (\mathrm{E}(p_j))^2}{p(1-p)} \\ &= \frac{\mathrm{var}(p_j)}{\mathrm{var}(p_{ij})} \\ &= \frac{\mathrm{var}(p_j)}{\overline{\mathrm{var}}(p_{ij}) + \mathrm{var}(p_j)}. \end{aligned}$$

R_V measures the difference between the likelihood for an A and an N to meet an A. Here an A receives help from another A more frequently than would occur by chance. This type of positive assortment is the key for altruism to evolve. Kin-based altruism, reciprocal altruism and network reciprocity all have this feature of positive assortment [2].

Here whenever $R_V > \frac{1}{r}$, the fraction of altruists in the population p will increase. At the equilibrium we will have p^* that satisfies

$$F_{ST}^* = \frac{1}{r}.$$

In summary, the existence of multilevel selection and the between-group variance make it likely for altruism to evolve.

Effect of the population size

In our model, we have a wide range of conditional strategies (27 in total). One potential concern is whether the population size (400) is large enough to allow for interactions between this

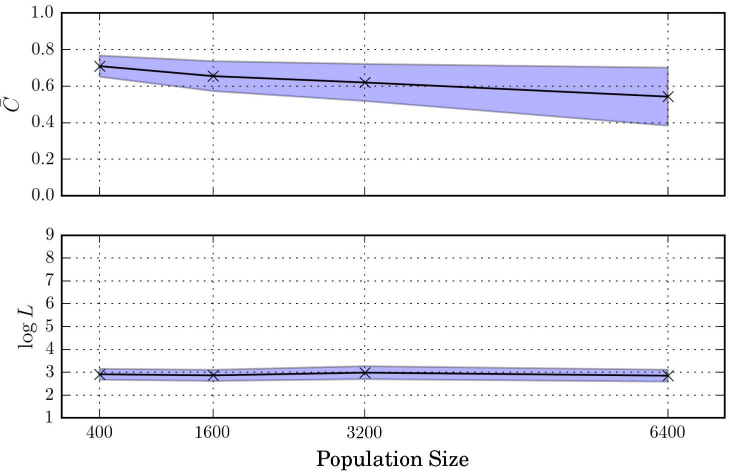

FIG. 1: Average cooperation level $\bar{C}$ and the logarithm of the average length of cooperative epochs $\log(L)$ for different population sizes while keeping the group size fixed at 20. Shaded area represents the corresponding 95% confidence intervals. Increasing the population size does not change the overall cooperation level or the stability of cooperation. Data is the average over 20 runs of simulation, each with 5000 generations.

large number of strategies. To test the robustness of the results to population size, we run simulations for different population size (400,1600,3200,6400) while keeping the group size fixed at 20. Figure 1 and Figure 2 demonstrate the overall cooperation level as well as distributions of strategies for different population sizes. The results are obtained from 20 runs of simulation, each with 5000 generations. As we can see from the figure, the overall cooperation levels and the strategy distributions do not demonstrate big differences for different population size, indicating that the population size 400 is sufficiently large for investigating the evolution of 27 strategies.

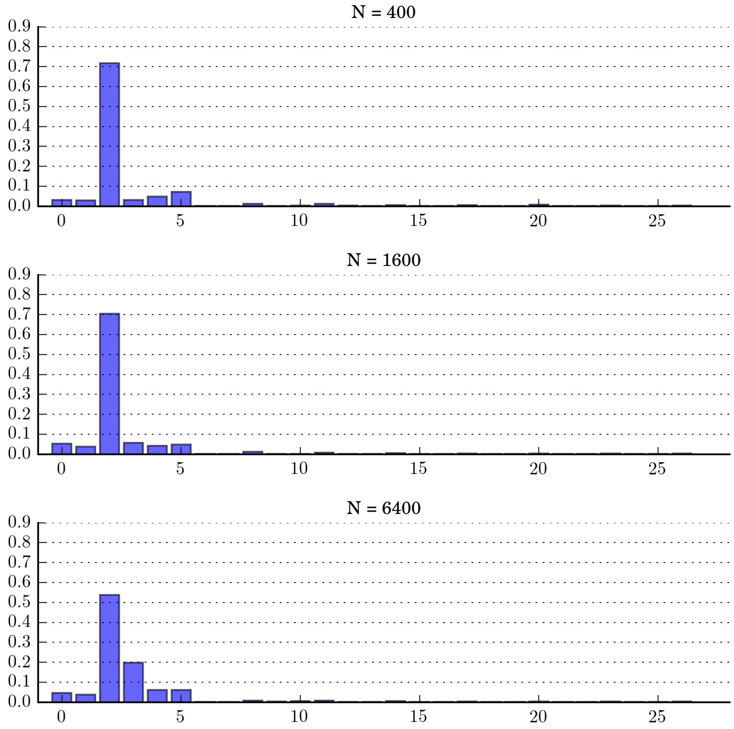

FIG. 2: Strategy distribution for different population sizes while keeping the group size fixed at 20. We do not oberve dramatic change in the distributions of strategies. Data is the average over 20 runs of simulation, each with 5000 generations.

[1] Fisher, R. A. *The Genetical Theory of Natural Selection* (Clarendon Press, Oxford, 1930).

[2] Bowles, S. and Gintis, H. *A Cooperative Species: Human Reciprocity and Its Evolution* (Princeton University Press, Princeton, 2011).
